# Evaluating the prognostic significance of p53 and TP53 mutations in HPV-negative hypopharyngeal carcinoma patients: a 5-year follow-up retrospective study

**DOI:** 10.1186/s12885-023-10775-9

**Published:** 2023-04-06

**Authors:** Qiang Huang, Feiran Li, Mengyou Ji, Lan Lin, Chunyan Hu

**Affiliations:** 1 Department of Otorhinolaryngology, Eye & ENT Hospital, Fudan University, Shanghai, 200031 China; 2 Department of Pathology, Eye & ENT Hospital, Fudan University, Shanghai, 200031 China

**Keywords:** Human papillomavirus, TP53, Exon mutation, Hypopharyngeal squamous cell carcinoma patients, Non-disruptive mutation

## Abstract

**Purpose:**

To evaluate prognostic significance of human papillomavirus (HPV) in hypopharyngeal squamous cell carcinoma patients, and to investigate the effect of p53 and TP53 mutations on the prognosis of patients.

**Methods:**

A total of 111 patients were enrolled in our retrospective study. HPV infection status was detected in formalin-fixed paraffin-embedded tissue by real-time multiplex PCR test. p53 expression was evaluate by immunohistochemical staining. TP53 exon mutations were analyzed by PCR amplification and Sanger sequencing. HPV infection status, p53 expression and TP53 mutation were compared with clinical outcome including overall survival and recurrence-free survival by Kaplan-Meier method and Log-rank test.

**Results:**

Of the 111 investigated patients, 18 (16.22%) were positive for HPV infection. HPV(-) patients have a worse clinical outcome than HPV(+) patients. TP53 mutations have similar mutation rates in patients with and without HPV (55.56% vs. 41.94%). p53 and TP53 mutation were not associated with prognosis of patients in HPV(-) patients. TP53 disruptive mutations were found both in patients with or without HPV infection. Furthermore, TP53 non-disruptive mutation had a significantly better clinical outcome than those with disruptive mutation in HPV(-) patients.

**Conclusion:**

Our results showed that HPV infection status is a strong prognostic indicator of survival. p53 and TP53 mutations do not appear to significantly impact survival in HPV(-) patients. TP53 disruptive mutation is associated with reduced survival in HPV(-)/TP53 mutation patients.

## Introduction

Head and neck squamous cell carcinoma (HNSCC) is one of the most common cancers in the world which is heterogeneous in tumor site, pathogenesis and cause with 890,000 new cases and 450,000 deaths in 2018 [1]. Hypopharyngeal squamous cell carcinoma (HPSCC) is a subtype of HNSCC, which 70% to 85% of cases are already in advanced stage when diagnosed due to the hidden location of the onset [2, 3]. The pathogenesis of HPSCC is multifactorial and is linked to smoking, alcohol consumption and infection with the human papillomavirus (HPV) [4–6]. Multimodality therapies, including surgery, chemotherapy, biologic therapy, and radiotherapy, particularly intensity-modulated radiotherapy (IMRT), are the current treatments for patients [7]. Despite recent advances in these treatment modalities, the overall survival remains poor over the past years.

Although HPV infection status has been identified as a risk factor in HNSCC, especially oropharyngeal squamous cell carcinoma (OPSCC) [8], prognostic significance of HPV in non-OPSCC HNSCC is still disputable [9]. Li et al. showed that HPV status was the greatest factor in survival outcome between the HPV-positive and -negative cohorts at the hypopharynx subsites through a large-sample analysis [10]. However, several studied reported that HPV does not appear to significantly impact survival or disease control in HPSCC patients [11–16].

In addition, the abrogation of p53 function is one of the most common molecular alterations in HNSCC [17, 18] through the mutation of its gene, TP53 [4], the loss of heterozygosity of TP53 [6] or interaction with viral proteins [19]. The involvement of p53 in apoptosis and cell cycle control, making it a reasonable prognostic biomarker [18]. In addition, the TP53 mutation profile observed in tumor samples suggests that these mutations differ in their impact on prognosis [20, 17]. The role of p53 or TP53 as a prognostic marker of HNSCC is controversial. As compared with wild-type TP53, the presence of any TP53 mutation was associated with decreased overall survival in HNSCC patients [20]. However, the results were inconsistent for specific HNSCC subtypes. Singh et al. reported that survival of oral squamous cell carcinoma (OSCC) patients was not affected by HPV and p53 status [21]. Moreover, Hong et al. did not show any evidence that p53 mutation could modify the effect of HPV status on outcomes from their study [22]. For now, no relation between HPV infection status, TP53 mutation and prognosis of HPSCC patients is currently well established.

Herein, we sought to evaluate the effect of HPV status on HPSCC survival, to determine the incidence of TP53 mutation in HPSCC and to seek associations among TP53 status, HPV status and survival.

### Methods and materials

#### Patient tissue and ethics approval

A cohort of 111 formalin fixed paraffin-embedded (FFPE) HPSCC tissues were collected from patients diagnosed with HPSCC pathologically after surgery in the Department of Otorhinolaryngology, Eye & ENT Hospital of Fudan University from January 2015 to January 2018. All participants provided written informed consent forms. This study was reviewed and approved by the Ethics Committee of Eye & ENT Hospital of Fudan University (No. 2018036).

### Detection of HPV genotype

Detection of HPV genotypes were analyzed by real-time polymerase chain reaction (PCR) using formalin fixed paraffin-embedded (FFPE) HPSCC tumor samples. Briefly, after deparaffinization and rehydration, DNA was isolated from FFPE tissue using QIAamp DNA FFPE Tissue Kit (Qiagen, Valencia, CA, USA) in accordance with the manufacturer’s instructions. Real-time PCR amplifications were performed in a Thermal Cycler (ABI 7500 Real-Time PCR System, Life Technologies, Shanghai, China) using HPV Genotyping Real-time PCR Kit (Hybribio Limited, China) which is a real-time multiplex PCR test for the detection of 23 HPV genotypes (HPV6, 11, 16, 18, 31, 33, 35, 39, 42, 43, 44, 45, 51, 52, 53, 56, 58, 59, 66, 68, 73, 81 and 82), in accordance with the manufacturer’s instructions [23, 24]. An HPV-positive tumor was defined as a tumor for which there was specific positive amplification of either HPV subtype.

### Immunohistochemical (IHC) staining and assessment

Tumor p53 protein expression was evaluated by means of immunohistochemical analysis with a mouse monoclonal antibody (1:200, Gene Tech, Shanghai) visualized with use of BenchMark Autostainer (Ventana Medical Systems, Tucson, USA). Positive p53 expression was defined as strong and diffuse nuclear staining. All sections were graded from level 0 to level 4 according to the following assessment: level 0, less than 1% positive cells; level 1, 1-9% positive cells; level 2, 10–49% positive cells; and level 3, ≥50% positive cells. Level 0 to level 1 was defined as low expression, and level 2 to level 3 was defined as high expression. The staining results were checked independently by two senior pathologists, and the discrepancies in immunostaining reviewing were solved by consensus.

### Database information

The Gene_Outcome module in TIMER2.0 (http://timer.cistrome.org/) [25] was used to evaluate the outcome significance of TP53 gene expression.

### TP53 mutation analysis

DNA extracted from FFPE tissue was used to analyze mutations in the TP53 gene. Three sets of primers were used to amplify genomic DNA sequences of exons 5, 6 and 8 with the most frequent TP53 mutations (Table 1). The PCR was conducted in a 20 μl reaction mix containing 1 μl of DNA, 10 μl of PremixTaq polymerase (Vazyme), 7 μl of ddH_2_O, and 2 μl of primers. The thermal cycling conditions were an initial denaturation at 94 ℃ for 5 min, followed by 28 cycles of denaturation at 94 ℃ for 30 seconds, primer annealing at 58 ℃ for 30 seconds, and extension at 72 ℃ for 1 min, and then a final extension at 72 ℃ for 10 min. The PCR products were analyzed by electrophoresis using a 2% agarose gel. PCR products were purified with Universal DNA Purification Kit (Tiangen Biotech), and submitted to a commercial company for Sanger sequencing. Sequencing was performed using ABI 3730 XL sequencer (Applied Biosystems). Sequencing results were analyzed with DNA Sequencing analysis software, interpreted with Sequencing analysis 5.2.0 software, and compared with Sequencher 5.1 software package.

**Table 1:** TP53 exon primer for detection

Target	Forward primer (5’-3’)	Reverse primer (5’-3’)	Product (bp)
Exon 5	TGTTTGTTTCTTTGCTGCCGT	CATCCAAATACTCCACACGCA	416
Exon 6	GCAGTCACAGCACATGACGGA	AATAAGCAGCAGGAGAAAGCC	360
Exon 8	AAGGGTGGTTGGGAGTAGATG	AATATTCTCCATCCAGTGGTTTC	391

Polymorphisms and mutations were determined based on the reference sequences available from the International Agency for Research on Cancer (IARC) TP53 database (http://p53.iarc.fr). The genetic mutations were described in accordance with the nomenclature rules of the Human Genome Variation Society (http://www.HGVS.org/varnomen). The hotspot mutation of TP53 was confirmed according to the TP53 Database (R20, July 2019, https://tp53.isb-cgc.org). TP53 mutations were grouped as ‘‘disruptive’’ and ‘‘non-disruptive’’ according to available information about the functional differences of various TP53 mutations [20]. Disruptive mutations were defined as stop mutations, frameshift mutations, or nonconservative mutations occurring within the key DNA-binding domain L2/L3. All other mutations were defined as non-disruptive mutations (excluding stop codons) [26].

### Statistical analysis

The primary endpoint was overall survival (OS), defined as the time from diagnosis to death. Secondary end point was recurrence-free survival (RFS), defined as the time from diagnosis to death or the first documented relapse. The Chi-square test was used to compare demographic and clinicopathologic characteristics. Statistical analysis was performed using IBM SPSS Statistics (version 22.0; IBM, Armonk, NY, USA), and graphed using GraphPad Prism (version 8; GraphPad Software, La Jolla, CA). OS and RFS were evaluated using the Kaplan-Meier method and Log-rank (Mantel-Cox) test. Differences were considered significant if the *p* value was <0.05.

## Results

### Overview of main clinical features of the cohort and prevalence of HPV

Collectively, a cohort of consecutive 111 HPSCC patients was included in the current study from January 2015 to January 2018 in Eye & ENT Hospital, Fudan University. The tumors mainly originated from the pyriform sinus (*n*=97, 87.39%), followed by the postcricoid region (*n*=7, 6.31%) and the posterior pharyngeal region (*n*=7, 6.31%). According to the 8^th^ AJCC staging system, most of patients were in advanced stage (104/111, 93.69%). During the 5-year follow-up period, 36.94% (41/111) patients developed regional tumor recurrence. The 5-year OS and RFS rate of the cohort was 65.77% and 63.06%, respectively. The mean OS time and RFS time was 40.85±17.40 and 37.36±19.59 months, respectively.

**Fig. 1:**
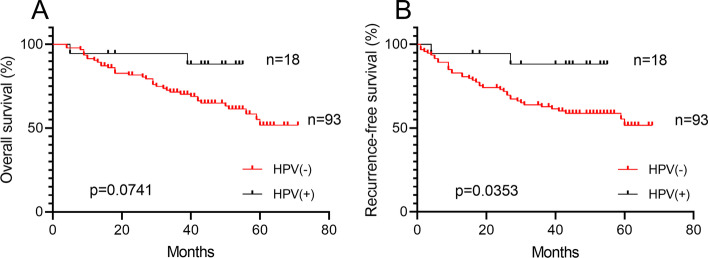
Prognostic significance of HPV infection status in 111 advanced HPSCC patients Kaplan-Meier estimates of (**A**) OS and (**B**) RFS among the 18 HPV(+) HPSCC patients (of whom 2 died) and the 93 HPV(-) HPSCC patients (of whom36 died). The mean OS and RFS among HPV(+) patients were 41.06 and 39.78 years, as compared with 40.81 and 36.89 years among HPV(-) patients. *p*=0.0741 for OS, *p*=0.0353 for RFS (Log-rank test)

Of the 111 investigated patients, 18 (16.22%) were positive for HPV infection (all types combined). Among them, HPV16 was the most prevalent subtype which accounted for 77.78%. In addition, there was 1 case of HPV56, 6 and 82 subtype, respectively. Of note, there was 1 case of HPV81 and 58 subtype double infection. By using Kaplan-Meier method, although it did not reach statistical significance, HPV(+) HPSCC patients had better OS than those of the HPV(-) patients (88.89 % vs 61.29%, *p*=0.0741, Fig. 1A). Furthermore, HPV(+) HPSCC patients had significant better RFS than those of the HPV(-) patients (88.89 % vs 58.06%, *p*=0.0353, Fig. 1B).

### Prognostic significance of p53 protein and TP53 gene expression in HPV-negative advanced HPSCC

**Fig. 2:**
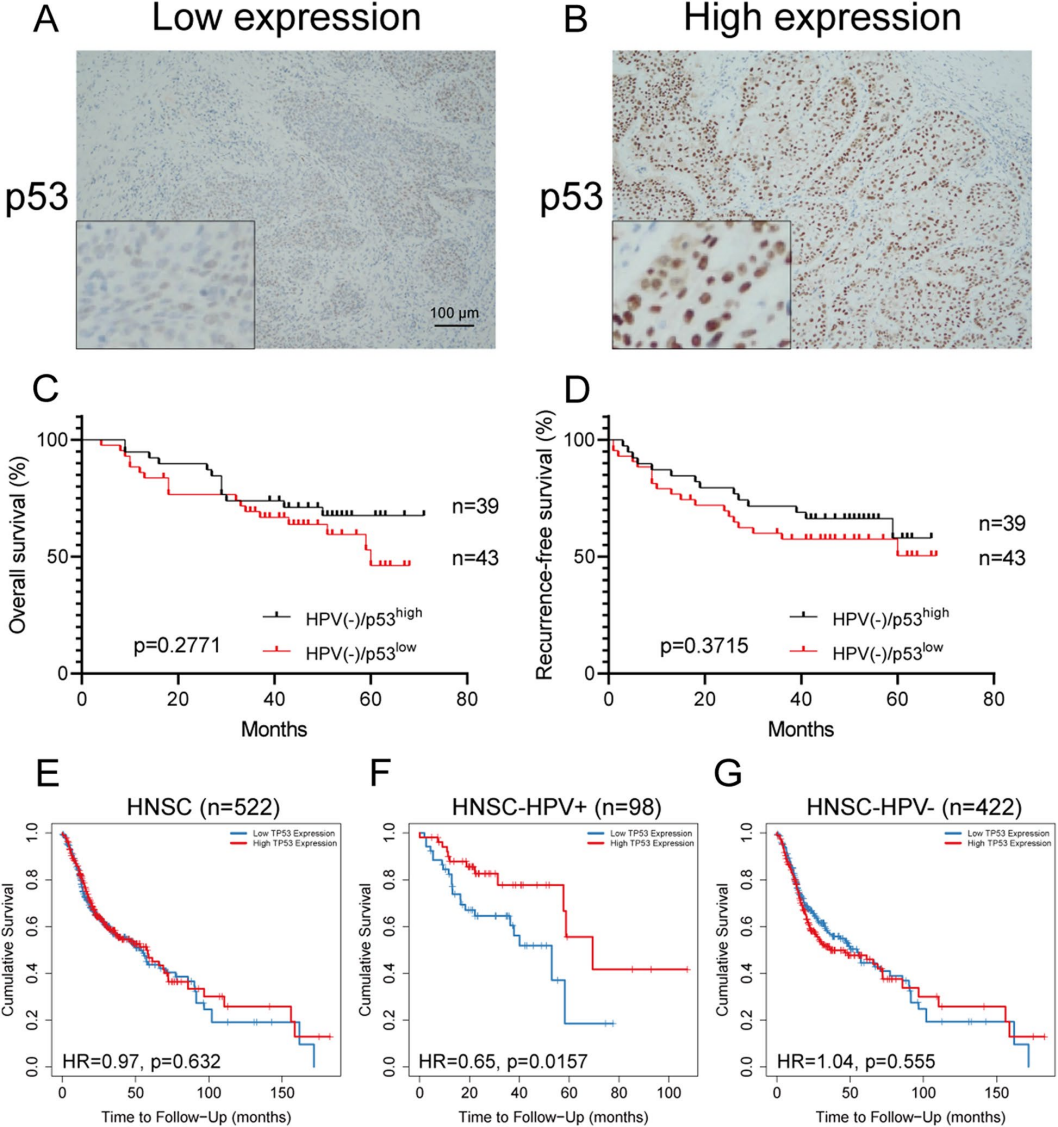
Patterns of p53 protein expression and prognostic significance of p53 protein and TP53 gene in HPV(-) advanced HPSCC patients (**A**) Complete absence or low expression (<50% of the tumour cells) of staining in the tumour. **B** Uniform strong nuclear staining in at least 50% of the tumour cells. Scale bar: 100 μm. Kaplan-Meier estimates of (**C**) OS and (**D**) RFS among the 39 HPSCC patients with HPV(-)/p53^high^ (of whom 12 died) and the 43 HPSCC patients with HPV(-)/p53^low^ (of whom 18 died). The mean OS and RFS among patients with HPV(-)/p53^high^ were 44.46 and 41.33 years, as compared with 39.86 and 35.63 years among patients with HPV(-)/p53^low^. *p*=0.2771 for OS, *p*=0.3715 for RFS (Log-rank test). Eleven patients did not undergo IHC staining due to sample reasons. **E** Kaplan-Meier curves shows outcome significance of TP53 gene expression among 522 head and neck cancer (HR=0.97, *p*=0.632). **F** Kaplan-Meier curves shows outcome significance of TP53 gene expression among 98 HPV(+) head and neck cancer (HR=0.65, *p*=0.0157). **G** Kaplan-Meier curves shows outcome significance of TP53 gene expression among 422 HPV(-) head and neck cancer (HR=1.04, *p*=0.555). Data available from Gene_Outcome module in TIMER2.0 (http://timer.cistrome.org/)

Since HPV(-) HPSCC patients have worse prognosis, we aimed to investigate whether tumor suppressor gene TP53 and its encoded p53 protein could be a prognostic indicators for HPV(-) HPSCC patients. By IHC staining, we found that most of p53 protein exist in the cellular nuclear of tumor cells, rather than in the stroma area (Fig. 2A-B). As shown in Fig. 2C-D, the results showed no statistical difference in OS and RFS between the HPV(-) patients with high and low expression of p53 protein (69.23% vs 58.14%, *p*=0.2771; 64.10% vs 55.81%, *p*=0.3715). Similarly, there was no difference in HPV(+) HPSCC patients (data not shown). Moreover, the expression level of p53 is not associated with the clinicopathologic features of HPSCC patients, regardless of HPV infection status (Table 2).

**Table 2:** Association between clinicopathological characteristics with the p53 staining in hypopharyngeal squamous cell carcinoma patients

Variable	p53 in total HPSCC (*n*=97)			p53 in HPV(+) HPSCC (*n*=15)			p53 in HPV(-) HPSCC (*n*=82)		
	Low	High	*p* value	Low	High	*p* value	Low	High	*p* value
**Age (years)**			0.9263			0.5804			0.6342
≤60	26	27		4	5		22	22	
>60	22	22		1	5		21	17	
**Smoking**			0.4495			>0.9999			0.6439
No	14	11		1	1		13	10	
Yes	34	38		4	9		30	29	
**Drinking**			0.9432			0.3333			0.6231
No	16	16		1	0		15	16	
Yes	32	33		4	10		27	23	
**Tumor site**			0.3502			>0.9999			0.3960
Pyriform sinus	40	44		4	8		36	36	
Not pyriform sinus	8	5		1	2		7	3	
**cT stage**			0.2651			0.3287			0.5071
T1-2	26	21		3	3		23	18	
T3-4	22	28		2	7		20	21	
**cN stage**			0.3756			0.5238			0.3083
N0	9	6		0	2		9	4	
N1-3	39	43		5	8		34	35	
**Clinical stage**			0.9811			>0.9999			0.6795
I+II	3	2		0	1		3	1	
III+IV	45	47		5	9		40	38	
**pT stage**			0.0528			0.0769			0.2484
T1-2	25	16		3	1		22	15	
T3-4	23	33		2	9		21	24	
**pN stage**			0.2133			0.5055			0.6044
N0	3	8		0	3		3	5	
N1-3	45	41		5	7		40	34	
**Pathological stage**			0.3772			>0.9999			0.2089
I+II+III	10	14		0	0		10	14	
IV	38	35		5	10		33	25	
**Tumor differentiation**			0.5045			>0.9999			0.6044
Well+ well-moderately	3	6		0	1		3	5	
Moderately+ Moderately-poorly	45	43		5	9		40	34	
**Surgical margin status**			0.4765			>0.9999			0.2700
≥0.5 cm	26	23		3	6		23	17	
<0.5 cm	22	26		2	4		20	24	
**Tumor size (cm)**			0.1135			0.5604			0.3098
≤3.5	33	26		2	2		31	24	
>3.5	15	23		3	8		12	15	
**Lymph nodal fusion**			0.5244			>0.9999			0.6136
No	37	35		3	6		34	29	
Yes	11	14		2	4		9	10	
**Metastatic lymph node size (cm)**			0.7256			>0.9999			0.9405
≤3	32	31		3	5		29	26	
>3	16	18		2	5		14	13	
**Cervical nodal necrosis**			0.9263			0.1009			0.7097
No	26	27		0	5		26	22	
Yes	22	22		5	5		17	17	
**Lymphovascular invasion**			0.9741			0.3333			0.8203
No	43	45		4	10		39	35	
Yes	5	4		1	0		4	4	
**Extracapsular spread**			0.2555			>0.9999			0.2963
No	39	35		3	6		36	29	
Yes	9	14		2	4		7	10	
**Fixation of hemilarynx**			0.4790			>0.9999			0.2368
No	25	22		4	8		21	14	
Yes	23	27		1	2		22	25	
**Thyroid gland invasion**			0.1173			>0.9999			0.2505
No	45	49		5	15		40	34	
Yes	3	0		0	0		3	0	
**Laryngeal invasion**			0.6188			0.6084			0.7539
No	21	19		3	4		18	15	
Yes	27	30		2	6		25	24	

Then, we aimed to evaluate the outcome significance of TP53 gene in head and neck cancer by online database analysis which is performed by Cox proportional hazard model (Fig. 2E-G). We found that the expression of TP53 is not an indicator of prognosis in total HNSCC and HPV(-) HNSCC patients (HR=0.97, *p*=0.632; HR=1.04, *p*=0.555), but is significant only in HPV(+) patients (HR=0.65, *p*=0.0157).

### Distribution and prognostic significance of TP53 mutation in HPV-negative advanced HPSCC

**Fig. 3:**
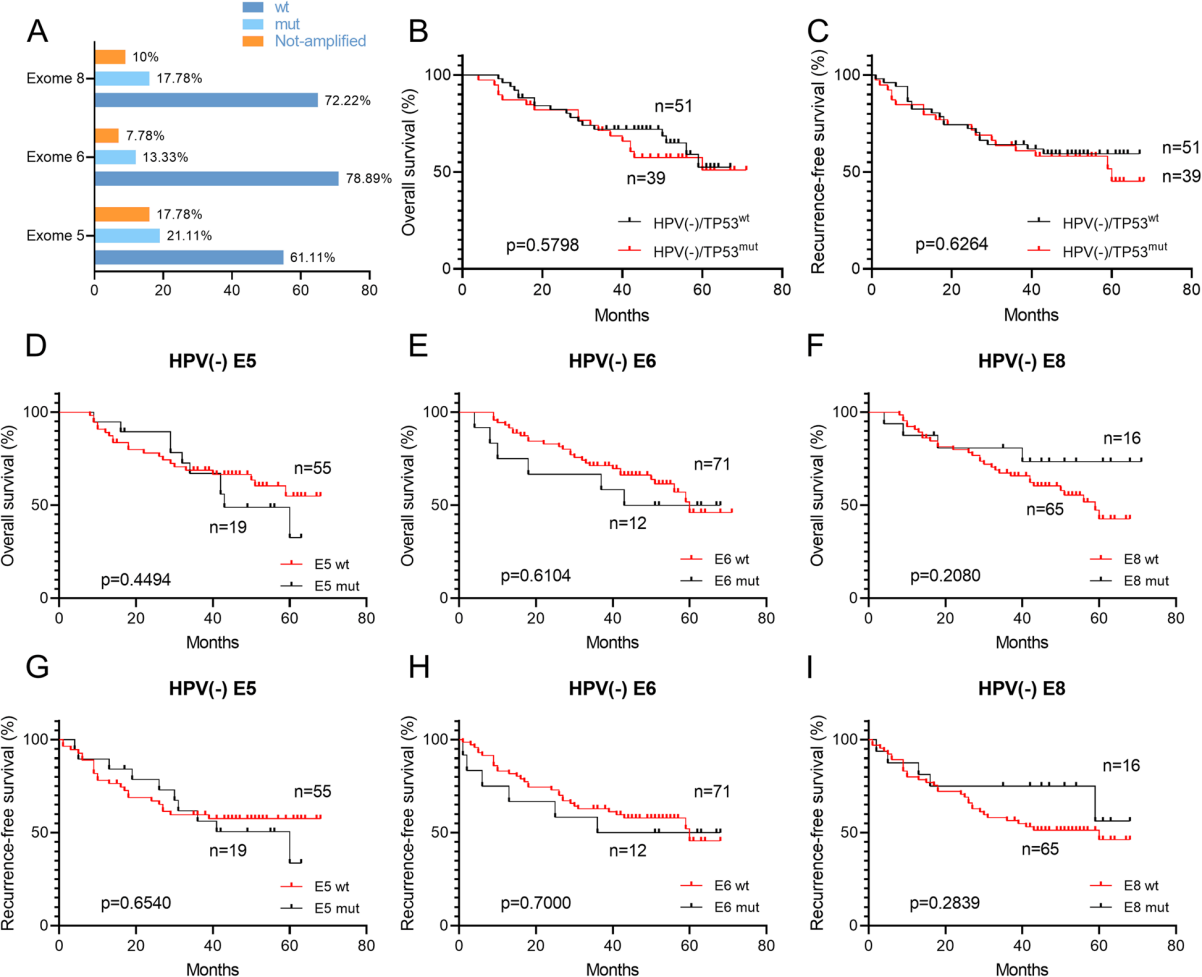
Distribution and prognostic significance of TP53 mutation in HPV(-) advanced HPSCC (**A**) Distribution of exon 5, 6 and 8 mutations of TP53. Kaplan-Meier estimates of (**B**) OS and (**C**) RFS among the 51 HPSCC patients with HPV(-)/TP53^wt^ (of whom 18 died) and the 39 HPSCC patients with HPV(-)/TP53^mut^ (of whom 17 died). The mean OS and RFS among patients with HPV(-)/TP53^wt^ were 41.10 and 36.78 years, as compared with 41.95 and 38.33 years among patients with HPV(-)/TP53^mut^. *p*=0.5798 for OS, *p*=0.6264 for RFS (Log-rank test). Three patients did not undergo TP53 mutation detection due to sample reasons. wt, wild type; mut, mutation. OS (**D-F**) and RFS (**G-I**) of each exon among the HPV(-)HPSCC patients. E, exon

Due to frequent mutation of TP53 in HNSCC patients, we then aimed to estimate the outcome significance of TP53 exon mutation. It was found that 41.94% (39/93) HPV(-) HPSCC patients had TP53 exon mutation (Fig. 3A). Among them, exon 5 had the most mutation events (21.11%), followed by exon 8 and exon 6 (17.78% and 13.33%). The mutation of any TP53 exon was defined as TP53 mutation, and we evaluated the significance of TP53 mutation for the prognosis of patients. However, there was no statistical difference in OS and RFS between the HPV(-) patients with wild and mutation TP53 (64.71% vs 56.41%, *p*=0.5798; 60.78% vs 53.85%, *p*=0.6264; Fig. 3B-C). Furthermore, to reduce the interference factor of index mixing, the outcome significance of every single exon mutation was evaluated. Unfortunately, not a single exon mutation was associated with prognosis in patients with HPV(-) HPSCC (*p*>0.05; Fig. 3D-I). Similarly, there was no difference in HPV(+) HPSCC patients (data not shown).

### Detailed TP53 exon mutation events and its prognostic significance in HPV-negative advanced HPSCC

**Fig. 4:**
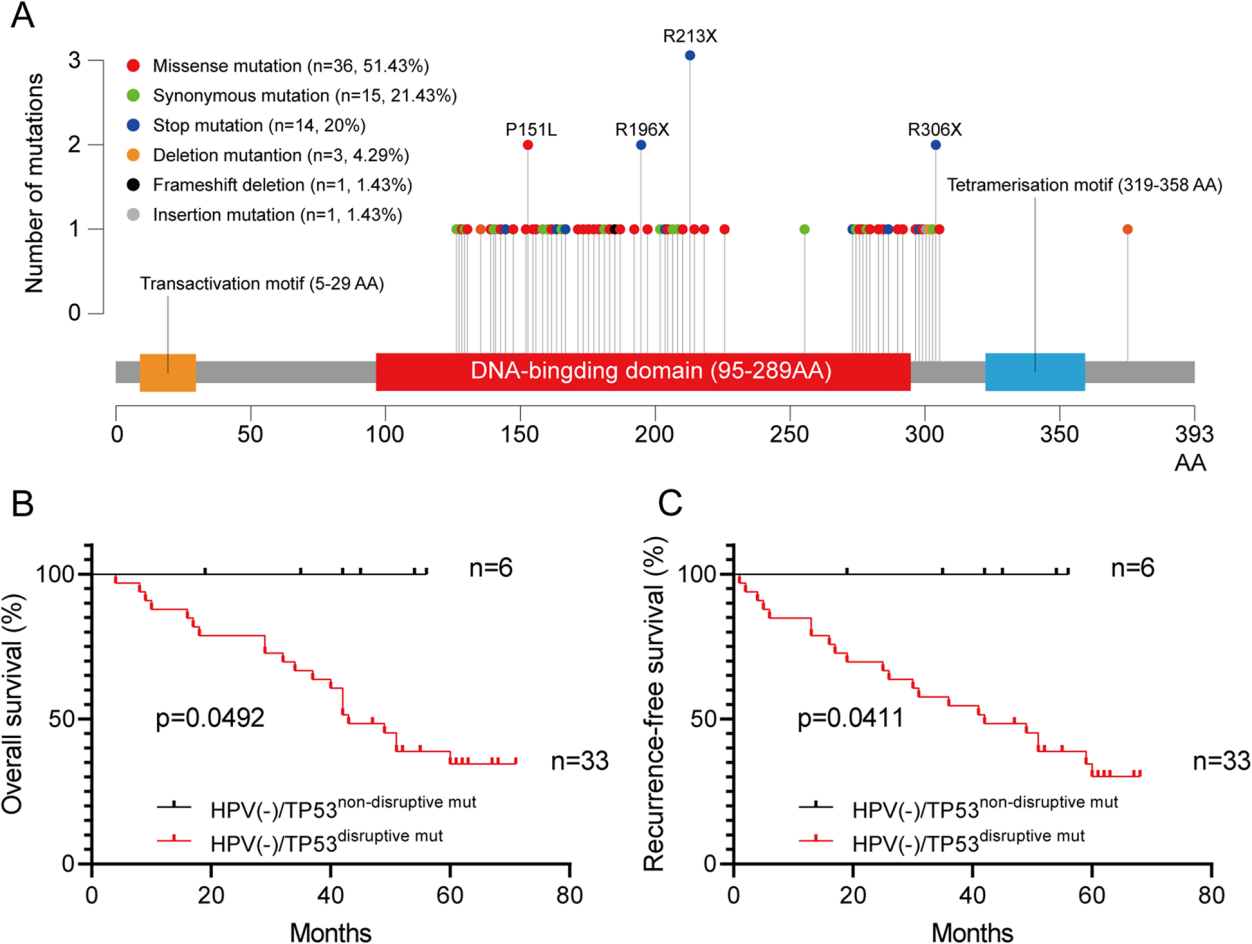
Distribution of TP53 exon mutation events and prognostic significance of disruptive mutation of TP53 in HPSCC patients (**A**) Histogram of number of cases with TP53 mutations by TP53 codon position and the top 4 most frequently mutated TP53 codons located in the DNA-binding domain in HPSCC patients. Kaplan-Meier estimates of (**B**) OS and (**C**) RFS among the 33 HPSCC patients with HPV(-)/TP53^disruptive mut^ and the 6 HPSCC patients with HPV(-)/TP53^non-disruptive mut^. *p*=0.0492 for OS, *p*=0.0411 for RFS (Log-rank test)

**Fig. 5:**
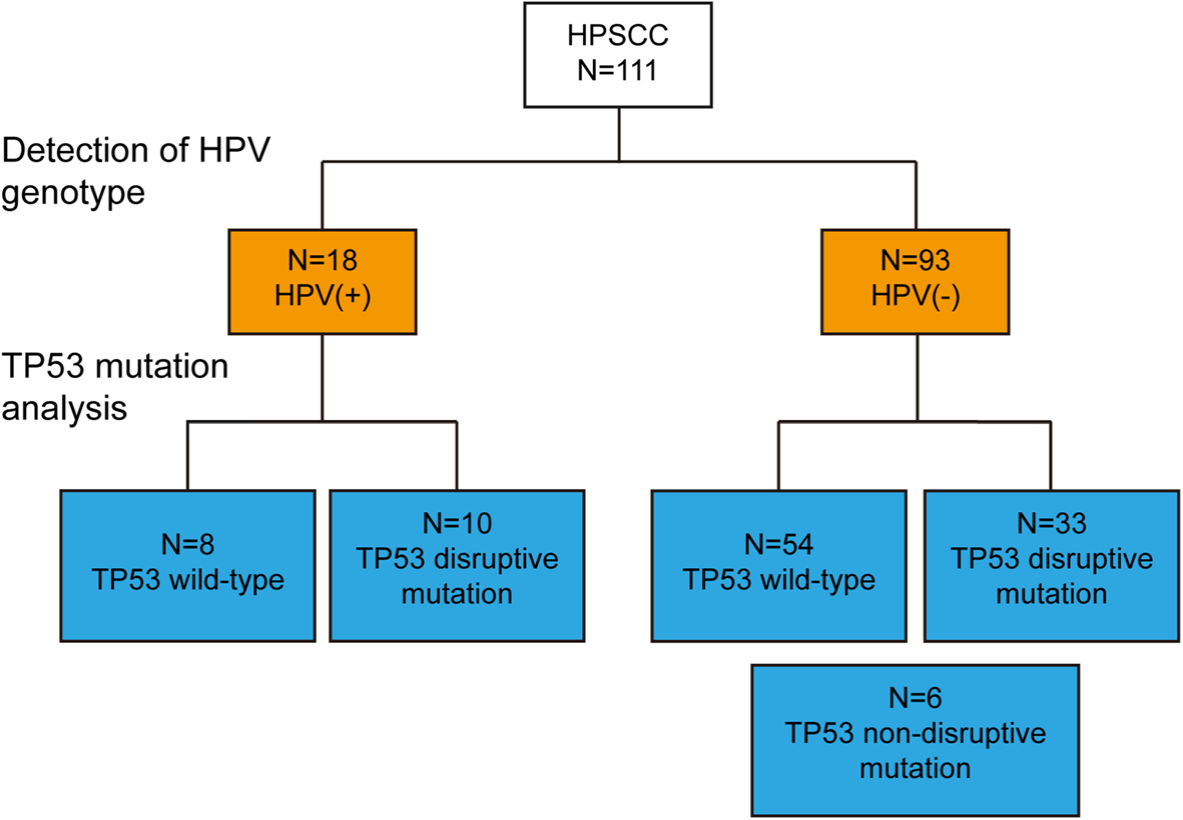
Flow diagram of the HPV and TP53 status results from 111 HPSCC patients

Altogether, 65 different types of variants (70 times) of TP53 were found in this study (Table 3), of which 47 variants have been reported as hot-spot mutations in previous studies. C:G > T:A was the most frequent pattern of substitution observed across all subject groups. Nearly half (36/70, 51.43%) of the variants were missense mutation, and 14 of 70 times (20%) resulted in stop codon. The location and amino acid changes are shown in Table 4. The most frequently found variants, detected in 111 subjects, was located at 637 codon in exon 6 resulting in a stop codon change (3 times), followed by variants at codon 452 in exon 5 (proline-to-leucine), and variants at codon 586 and 916 in exon 6 and 8 resulting in a stop codon change (2 times). The majority (36/49, 73.47%) of subjects with TP53 mutations had a mutation at only one spot, while one subject had mutations at four spots, four subjects had mutations at three spots and eight subjects had mutations at two spots (Fig. 4A). Furthermore, the proportion of patients with TP53 mutation was similar among HPSCC cases with or without HPV infection (10/18, 55.56% vs. 39/93, 41.94%, *p*=0.2868, Fig. 5).

**Table 3:** Overview of TP53 mutation events in 39 patients with TP53 mutation

Exon	Total	Missense mutation	Synonymous mutation	Stop mutation	Deletion mutation	Insertion mutation	Frameshift deletion
E5	31	17	9	3	2	0	0
E6	18	9	2	6	0	0	1
E8	21	10	4	5	1	1	0

**Table 4:** TP53 mutations found from study subjects

**Frequency of detection**	**Exon**	**Codon**	**Base substitution**	**Amino acid change**	**Mutation Type**	**Within DNA binding Domain**	**Hotspot**^**a**^
1	E05	387	CT	A129A	Heterozygous	Yes	Not detected
1	E05	413	CT	A138V	Heterozygous	Yes	Yes
1	E05	477	CT	A159A	Heterozygous	Yes	Yes
1	E06	572_574	delCTC	A189A	Homozygous	Yes	Yes
1	E06	566	CT	A189V	Heterozygous	Yes	No
1	E08	919	GA	A307T	Heterozygous	No	Not detected
1	E05	405	CT	C135C	Heterozygous	Yes	Yes
1	E05	404	GA	C135Y	Heterozygous	Yes	Yes
1	E05	423_427	delCCCTG	C140Cfs*6	Heterozygous	Yes	No
1	E05	423	CT	C141C	Heterozygous	Yes	Yes
1	E05	527	GT	C176F	Heterozygous	Yes	Yes
1	E05	442	GT	D148Y	Heterozygous	Yes	No
1	E06	624	CT	D208D	Heterozygous	Yes	Yes
1	E06	592	GA	E198K	Heterozygous	Yes	Yes
1	E06	610	GT	E204X	Heterozygous	Yes	Yes
1	E08	811	GT	E271X	Heterozygous	Yes	No
1	E08	813	GA	E271E	Heterozygous	Yes	Yes
1	E08	811	GA	E271K	Heterozygous	Yes	Yes
1	E08	854	AG	E285G	Homozygous	Yes	No
1	E08	856	GA	E286K	Heterozygous	Yes	No
1	E08	859	GT	E287X	Heterozygous	Yes	Yes
1	E08	880	GT	E294X	Heterozygous	No	Yes
1	E08	892	GA	E298K	Heterozygous	No	Yes
1	E05	461	GA	G154D	Heterozygous	Yes	Yes
1	E05	534	CT	H178H	Heterozygous	Yes	Yes
1	E05	536	AG	H179R	Heterozygous	Yes	Yes
1	E05	486	CT	I162I	Heterozygous	Yes	Yes
1	E05	485	TC	I162T	Heterozygous	Yes	No
1	E06	583	AT	I195F	Heterozygous	Yes	Yes
1	E08	915	GA	K305K	Homozygous	No	Yes
1	E08	866	TC	L289P	Homozygous	Yes	No
1	E08	865	CG	L289V	Heterozygous	Yes	No
1	E05	426	TC	P142P	Heterozygous	Yes	Yes
1	E05	452	CA	P151H	Heterozygous	Yes	Yes
2	E05	452	CT	P151L	Heterozygous	Yes	Yes
1	E05	457	CT	P153S	Heterozygous	Yes	No
1	E05	530	CT	P177L	Heterozygous	Yes	No
1	E06	667	CT	P223S	Heterozygous	Yes	Yes
1	E05	408	AG	Q136Q	Homozygous	Yes	Yes
1	E05	430	CT	Q144X	Homozygous	Yes	No
1	E05	493	CT	Q165X	Heterozygous	Yes	No
1	E05	523	CT	R175C	Heterozygous	Yes	Yes
1	E05	524	GA	R175H	Heterozygous	Yes	Yes
2	E06	586	CT	R196X	Heterozygous	Yes	Yes
1	E06	626	GA	R209K	Heterozygous	Yes	Yes
3	E06	637	CT	R213X	Heterozygous	Yes	No
1	E06	638	GT	R213L	Heterozygous	Yes	Yes
1	E08	801	GA	R267R	Heterozygous	Yes	Yes
1	E08	846	GA	R282R	Heterozygous	Yes	Yes
1	E08	844	CT	R282W	Homozygous	Yes	Yes
1	E08	869	GT	R290L	Heterozygous	No	Yes
2	E08	916	CT	R306X	Heterozygous	No	Yes
1	E05	419	CT	T140I	Heterozygous	Yes	No
1	E06	632	CT	T211I	Heterozygous	Yes	Yes
1	E08	919	delG	T304P	Heterozygous	No	No
1	E05	428	TC	V143A	Heterozygous	Yes	No
1	E05	517	GT	V173L	Heterozygous	Yes	Yes
1	E06	609	GA	V203V	Homozygous	Yes	Yes
1	E08	814	GA	V272M	Heterozygous	Yes	Yes
1	E05	489	CA	Y163X	Heterozygous	Yes	No
1	E05	489	CT	Y163Y	Heterozygous	Yes	Yes
1	E06	613	TC	Y205H	Heterozygous	Yes	Yes
1	E06	659	AG	Y220C	Heterozygous	Yes	Yes
1	E05	376-28_413	delCAACTCTGTCTCCTTCCTCTTCCTACAGTACTCCCCTGCCCTCAACAAGATGTTTTGCCAACTGGC		Heterozygous	No	Not detected
1	E08	894_895	insAG		Heterozygous	No	Yes

This form made in accordance with the nomenclature rules of the Human Genome Variation Society (http://www.HGVS.org/varnomen). del, deletion; ins, insertion; fs, frame shift

^a^ According to the TP53 Database (R20, July 2019): https://tp53.isb-cgc.org

As a transcription factor, p53 mutation in the domain of DNA binding region, which is called disruptive mutation, lead to loss of p53 protein DNA binding and gene expression regulation. We then aimed to estimate the outcome significance of disruptive mutation in HPV(-)/TP53^mut^ HPSCC patients. As shown in Fig. 4B-C, when compared with the patients with disruptive mutation (33 cases), we found that patients without disruptive mutation (6 cases) had a significantly better OS (51.52% vs. 83.33%, *p*=0.0492) and RFS (48.48% vs. 83.33%, *p*=0.0411).

## Discussion

In this current study, to address the controversial information regarding the role of HPV infection in HPSCC, the pathological tissues of 111 HPSCC patients were collected, and the relationship between HPV infection status and prognosis of HPSCC patients was proved by detecting HPV genotypes. The prognosis role of p53 in HPV(-) HPSSCC patients was also demonstrated by detecting the p53 protein content and comprehensive landscape of TP53 mutation.

First, we showed that HPV(-) HPSCC patients had worse OS and RFS than those of the HPV(+) patients, despite several reports to the contrary [11–15]. We speculated that this might be related to the differences in sample detection methods and the characteristics of the included population. The World Health Organization and the Union for International Cancer Control recommend the use of p16 IHC to simplify the detection of HPV infection in HNSCC, particularly in OPSCC [27, 28]. However, it is disadvantageous to use the gold standard to diagnose HPV infection in non-OPSCC HNSCC [9, 16, 29]. In this study, we performed p16 IHC staining and tried to detect HPV infection status (data not shown). However, only five of the 111 HPSCC samples were p16 positive (4.50%), and only two of them tested positive for HPV genotype detection. The harmonization of HPV testing method needs to be addressed, and the results of the analysis of different anatomical sites of HNSCC should be interpreted with caution before more large sample data are presented.

Secondly, although Hong et al. showed that HPV+ patients were significantly less likely to have p53 mutations than HPV-negative patients [22], our results showed that 41.94% (39/93) of HPV(-) HPSCC patients developed TP53 mutations, while 55.56% (10/18) of HPV(+) HPSCC patients developed TP53 mutations (*p*=0.287). Moreover, although previous study [30] found the significant correlation between a high expression of p53 and a histological grade of well differentiation, advanced tumor (T) and TNM stage in HPSCC patients, we found that p53 expression level, similarly like TP53 mutation, was not associated with prognosis of HPSCC patients, regardless of HPV infection status. We speculated that this is related to sample size differences and inconsistent antibodies used in IHC staining. Similar to our results, Singh et al. [21] and Hong et al. [22] found that survival of patients was not affected by p53 status. However, Ernoux-Neufcoeur et al. found that the 5-year disease-free survival rate was 73% in p53- HPSCC tissues versus 48% in p53+ HPSCC tissues[14]. This suggests that larger sample sizes and better postoperative follow-up are needed to clarify the role of controversial p53 status as an indicator of patient prognosis.

Finally, our dichotomous categorization based on protein folding and certain features of the gene classified HPV(-) patients into disruptive mutation and non-disruptive mutation groups, finding that patients with non-disruptive mutation had a significantly better OS and RFS than those with disruptive mutation. Several studies showed that the disruptive mutation is only found in HPV-negative HNSCC which suggest the absence of TP53 disruptive mutations may underlie the improved patient outcome of HPV-positive HNSCC [26, 31, 32]. However, TP53 mutations were found in 10 of the 18 HPV(+) HPSCC patients in our study, all of which were disruptive mutations. Considering the complexity of p53 interactions, the functional properties of each mutation may uniquely affect pathways for maintaining genomic integrity that involve p53. The biologic effects of TP53 mutations may also be influenced by the presence or absence of the remaining wild-type allele and by the gain of function of some mutants [20].

Overall, our results suggest that HPSCC patients without HPV have a worse clinical outcome than patients with HPV. TP53 mutations have similar mutation rates in HPSCC patients with and without HPV. Moreover, p53 and TP53 mutation were not associated with prognosis of HPSCC patients in HPV(-) HPSCC patients. TP53 disruptive mutations were found in HPSCC patients with or without HPV. Furthermore, TP53 non-disruptive mutation had a significantly better clinical outcome than those with disruptive mutation in HPV(-) HPSCC patients.

## Data Availability

The datasets generated and/or analyzed during the current study are not publicly available due to privacy and ethical issues, but are available from the corresponding author on reasonable request.
